# Tuberculosis-Associated Chronic Kidney Disease

**DOI:** 10.4269/ajtmh.2011.11-0014

**Published:** 2011-06-01

**Authors:** Jobson Lopes de Oliveira, Geraldo Bezerra da Silva Junior, Elizabeth De Francesco Daher

**Affiliations:** Division of Nephrology, Department of Internal Medicine, Federal University of Ceará, Fortaleza, Ceará, Brazil; School of Medicine, University of Fortaleza, Fortaleza, Ceará, Brazil

## Abstract

Extrapulmonary tuberculosis (TB) account for approximately 15–20% of TB cases in immunocompetent patients. The genitourinary system is the third most commonly affected site. We report the case of a 20-year-old man admitted with fever, chills, dry cough, right flank pain, and oliguria who developed renal function loss. The pyelogram evidenced silence of the right kidney, and the abdominal and pelvic magnetic resonance showed significant dilation of the right pyelocaliceal system and proximal ureter. Biopsies of renal cortex and retroperitoneal lymph nodes showed caseous granuloma consistent with TB. Treatment was started with rifampicin, isoniazid, pyrazinamide, and ethambutol, and the patient presented a favorable outcome but with non-dialytic chronic kidney disease. This case illustrates a case of chronic kidney disease secondary to TB in a young, otherwise healthy man.

A 20-year-old man was admitted with fever, chills, dry cough, right flank pain, and oliguria. Physical examination showed a palpable mass in the right flank. Laboratory analysis was notable for a creatinine clearance = 59 mL/min per 1.73 m^2^. Tuberculin skin test was 8 mm; human immunodeficiency virus (HIV) serology was negative. Urinalysis showed sterile pyuria, but microscopy showed acid fast bacilli. The plain abdominal X-ray film showed silence of the right kidney (Figure 1). The chest X-ray showed a fibrocavity infiltrate with small nodules in the right upper lung (Figure 2). The abdominal and pelvic magnetic resonance showed significant dilation of the right pyelocaliceal system and proximal ureter, with thickening of its middle one-third and amorphous retroperitoneal images in the pericaval space with maximum measures of 2.5 × 8.5 cm, consistent with grouped lymph nodes (Figure 3). Biopsies of the renal cortex and retroperitoneal lymph nodes showed caseous granulomas consistent with tuberculosis (TB). Treatment was started with rifampicin, isoniazid, pyrazinamide, and ethambutol, and the patient was left with chronic kidney disease (creatinine clearance after TB treatment = 61 mL/min per 1.73 m^2^). The genitourinary system is often affected in TB resulting from hematogenous spread from primary pulmonary TB.^1^ Urogenital TB is suspected particularly with sterile pyuria.^2^,^3^ Renal dysfunction may result from direct infection of the kidney parenchyma orureteral obstruction with resultant hydronephrosis.

**Figure 1.**
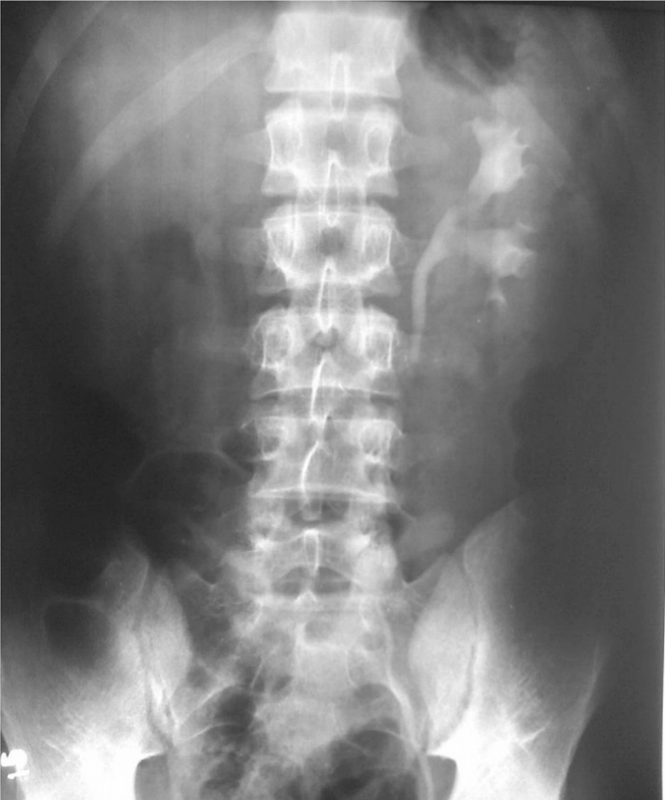
Pyelogram showing right kidney silence.

**Figure 2.**
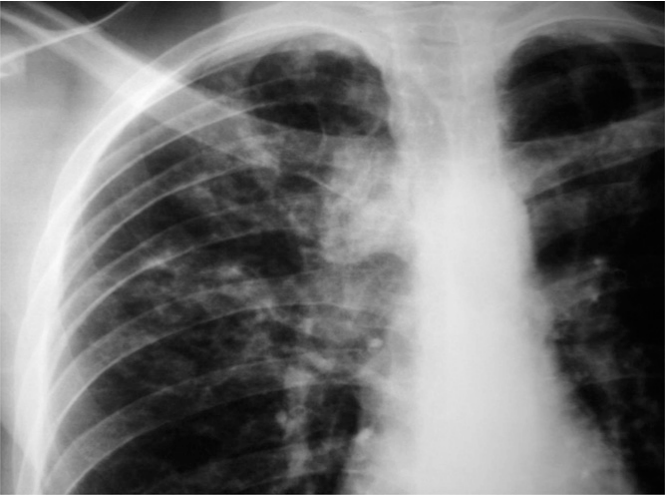
Chest X-ray showing small nodules scattered in the right lung.

**Figure 3.**
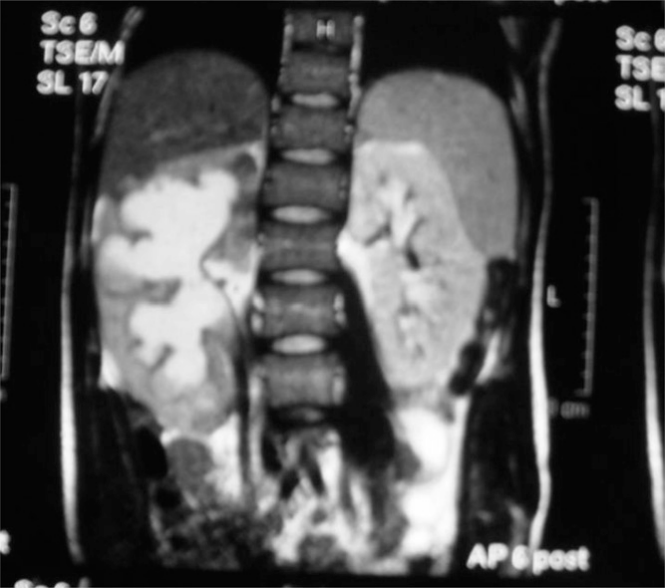
Nuclear magnetic resonance showing severe dilation of right pyelocaliceal system and proximal ureter.
